# Publisher Correction: A SARS-CoV-2 (COVID-19) biological network to find targets for drug repurposing

**DOI:** 10.1038/s41598-021-94440-w

**Published:** 2021-07-26

**Authors:** Mahnaz Habibi, Golnaz Taheri, Rosa Aghdam

**Affiliations:** 1 Department of Mathematics, Qazvin Branch, Islamic Azad University, Qazvin, Iran; 2 Department of Electrical Engineering and Computer Science, KTH Royal Institute of Technology, Stockholm, Sweden; 3 Science for Life Laboratory, Stockholm, Sweden; 4 School of Biological Sciences, Institute for Research in Fundamental Sciences (IPM), Tehran, Iran

Correction to: *Scientifc Reports* 10.1038/s41598-021-88427-w, published online 30 April 2021

In the original version of this Article author Mahnaz Habibi was incorrectly affiliated with ‘Science for Life Laboratory, Stockholm, Sweden.’ The correct affiliation is listed below.

Department of Mathematics, Qazvin Branch, Islamic Azad University, Qazvin, Iran.

Furthermore, an affiliation for author Golnaz Taheri was omitted. The additional affiliation is listed below:

Science for Life Laboratory, Stockholm, Sweden.

The original Article has been corrected.

